# Ubiquitin isopeptidase and esterase activities have distinct catalytic requirements

**DOI:** 10.64898/2026.10.20.760496

**Published:** 2026-10-21

**Authors:** Rachel E Lacoursiere, Paul P Geurink, Jonathan N Pruneda

## Abstract

Post-translational ubiquitin signaling is reversed by dedicated proteases termed deubiquitinases, many of which utilize a cysteine-based catalytic triad to cleave the isopeptide linkage. This activity represents canonical isopeptidase activity that has been mechanistically well characterized. By contrast, non-canonical ubiquitination, linked instead by an ester bond, is reversed by esterase activity through a relatively less well understood process. Several deubiquitinases, including examples from pathogenic bacteria and humans, have been shown to harbor dual isopeptidase and esterase activities. Here, we show that while both functions can take place within the same active site, they exhibit distinct catalytic requirements. In fact, the catalytic cysteine itself is not strictly required for ubiquitin esterase activity, as highlighted by substantial residual activities in both bacterial and human deubiquitinases following the typical Cys-to-Ala substitution. A Cys-to-Thr substitution, meanwhile, effectively eliminates both isopeptidase and esterase activity. Further illustrating distinct catalytic requirements, cryptic ubiquitin esterase activities were identified in OTUD6B and OTULINL, two reportedly inactive human (pseudo-) deubiquitinases. Overall, our work establishes new perspectives and approaches for studying the regulation of non-canonical ester-linked ubiquitination.

## Full Text Availability

The license terms selected by the author(s) for this preprint version do not permit archiving in PMC. The full text is available from the preprint server.

